# Scc*mec* type II gene is common among clinical isolates of methicillin-resistant *Staphylococcus aureus* in Jakarta, Indonesia

**DOI:** 10.1186/1756-0500-6-110

**Published:** 2013-03-23

**Authors:** Latre Buntaran, Mochammad Hatta, Andi R Sultan, Ressy Dwiyanti, Muhammad Sabir

**Affiliations:** 1 Department of Microbiology, RSAB Harapan Kita, Jakarta, Indonesia; 2 Department Medical Microbiology, Laboratory Molecular Biology and Immunology, Faculty of Medicine, Hasanuddin University, Makassar, Indonesia; 3 Department of Microbiology, Faculty of Medicine, Tadulako University, Palu, Indonesia

## Abstract

**Background:**

Community Acquired Methicillin Resistant *Staphylococcus aureus* (CA-MRSA) is a strain of MRSA that can cause infections in patients in the community, in which these patients had no previous risk factors for MRSA infection and the patient received 72 hours prior to infection when admitted to hospital. This study aims to determine and compare the characteristics of epidemiological, clinical, and molecular biology of CA-MRSA with HA-MRSA.

**Methods:**

A total of 11 clinical strains of Methicillin-resistant *Staphylococcus aureus* (MRSA) and Methicillin-sensitive *Stapylococcus aureus* (MSSA) were collected from 2 hospitals in Jakarta, Indonesia in 2012. SCC*mec* typing was performed by multiplex polymerase chain reaction (PCR) and the presence of six genes (*vraR, vraG, vraA, vraF,fruA*, and *fruB*) associated with vancomycin resistance was examined by simple PCR analysis.

**Results:**

We found three strains of community-acquired MRSA with SCC*mec* type II and one strain of hospital-acquired MRSA with SCC*mec* type IV. The other seven strains did not contain *mecA* genes and SCC*mec*. Plasmid pUB110 was found in one strain of community-acquired MRSA and two strains of hospital-acquired MRSA. *vraA* genes were present in 9 of the 11 strains, *vraF* in 4, *vraG* in 5, and *vraR* in 4. Note worthily, three quarters of strains without pUB110 contained *vraR* and *vraF*, and 70% contained *vraA*, whereas 60% of strains with pUB110 contained *vraG*.

**Conclusion:**

Based on these results, we should be concerned about the possibility of transition from MRSA strains sensitive to vancomycin in VISA strains of MRSA strains obtained in clinical trials. But first we need to look the existence of natural VISA or hVISA among these MRSA strains.

## Background

*Staphylococcus aureus* (*S. aureus*) causes a wide variety of infections with clinical symptoms ranging from mild skin infections to severe deep infections. One of the important strains frequently found among nosocomial infections is Methicillin-resistant *Staphylococcus aureus* (MRSA) [1]. Data from the previous study showed higher prevalence and variations of MRSA in countries of the Asia-Pacific region than in Europe [2]. In some countries, such as Korea, Hong Kong, and Japan, the prevalence even exceeded 70% of all S. aureus isolated from hospitalized patients [3-6].

MRSA is resistant to methicillin and other related β-lactam antibiotics, such as cefoxitin and oxacillin [1]. Initially, MRSA infections were associated only with infection exposure in health care and hospital settings, and were therefore referred to as Hospital-acquired MRSA (HA-MRSA) [7]. Two decades ago, Community-acquired MRSA (CA-MRSA) started to emerge among MRSA isolates from individuals with no or minimal exposure to health care facilities [8,9]. Currently, this strain tends to be more common among *S. aureus* infections as it is increasingly reported, particularly among children and young adults [8-11]. CA-MRSA strains are roughly classified into two main groups. The first group consists of CA-MRSA strains that are resistant to mono beta-lactam or beta-lactams and erythromycin and usually infect healthy patients who are not predisposed to MRSA [12]. The second group consists of MRSA strains isolated from individuals who have risk factors for infection [13]. Clinically, the CA-MRSA strains can be isolated from severe infections such as osteomyelitis, bacteremia, endocarditis, and pneumonia [14-17].

The rapid evolvement and continuous spread of new MRSA strains may due to their capability to acquire and to use antimicrobial resistance genes encoded by mobile genetic elements such as Staphylococcal cassette chromosome *mec* (SCC*mec*)[18-21]. SCC*mec* is a mobile genetic element which harbors the methicillin resistance gene *mecA*[22]. Based on *mec* and *ccr* gene complex variations, there are 11 SCC*mec* types have been described so far, and also some subtypes or sub variations have been identified [20,23-25]. Interestingly these genotype variations also reflected their antimicrobial characteristic [26]. SCC*mec* types I-III are associated with HA-MRSA isolates, while types IV and V have been found related to CA-MRSA [26,27]. A previous study reported that up to 80% of MRSA isolates were of sequence type 22-MRSA-SCC*mec* type IV (ST22-MRSA-IV) [28].

Several reports have indicated the possibility that the incidence of CA-MRSA infection would surpass that of HA-MRSA infection [16,29,30]. Considering the wide spread of CA-MRSA in Asian countries in particular, there is an urgent need of epidemiological or molecular studies of this strain to guide targeting of effective therapeutic agents. In the present study, therefore, we studied the molecular variation of MRSA isolates obtained from two hospitals in Jakarta in the year 2012. We found that SCC*mec* type II was the predominant SCC*mec* type among these clinical isolates*.* As the main therapy for MRSA, vancomycin may contribute to the emergence of a vancomycin-intermediate *S. aureus* (VISA) strain. As previously reported, VISA can emerge from a vancomycin susceptible *S. aureus* (VSSA) strain during chronic infection – but the genetic factors tcontributing to this phenomenon still need to be further defined [31-34]. Therefore we also studied certain VISA gene variations of these strains.

## Methods

### Bacterial strains

A total of 11 clinical strains of *S. aureus* were collected in 2012 from two hospitals in Jakarta: RSAB Harapan Kita and Siloam Kebun Jeruk, Indonesia (Table 1). Only one strain per patient was included. Isolates of *S.aureus* colonies were identified on the basis of pigments and clotting factors. Zone barriers were determined on Mueller-Hinton agar according to the Clinical and laboratory standards institute (CLSI) guidelines. Strains were incubated at 35º for 18 hours then the diameter of inhibition zone was determined. Amoxicillin clavulanate, cefuroxime, ceftriaxone, cefotaxime, ceftazidime, cefepime, imipenem, cotrimoxazole, clindamycin, amikacin, ciprofloxacin, levofloxacin, vancomysin, linezolid, teicoplanin, tigecyclin, and fosfomycin were tested. Breakpoint for the definition of antibiotic resistance in *S. aureus* was based on CDC guidelines manual.

**Table 1:** **Characteristics of clinical samples with SCC*mec *typing**, **plasmid pUB110 and the sensitivity of non beta-**lactam **antibiotics**

**Sample**	**Specimens**	***SCCmec***	**pUB 110**	**SXT**	**CC**	**AN**	**CIP**	**LVFX**	**VA**	**TEC**	**TGC**	**FOS**	**LZ**
4	Urine	IV	+	R	S	S	S	S	S	S	S	S	S
9	Sputum	II	+	R	R	S	R	R	S	S	S	R	S
11	Throat swab	II	+	R	R	S	R	R	S	S	S	R	S
10	Pus	II	-	R	R	S	R	R	S	S	S	R	S
1	Blood	-	-	S	S	S	S	S	S	S	S	S	S
2	Blood	-	-	S	S	S	S	S	S	S	S	S	S
3	Bronchial discharge	-	-	R	S	S	S	S	S	S	S	R	S
5	Blood	-	-	R	S	S	S	S	S	S	S	R	S
6	Urine	-	-	S	S	S	S	S	S	S	S	S	S
7	Urine	-	-	S	S	S	S	S	S	S	S	S	S
8	Urine	-	-	S	S	S	S	S	S	S	S		S

### Genomic DNA isolation

Total genomic DNA was isolated using the Wizard® genomic DNA purification kit (Promega corporation, Madison, WI, USA).

### Multiplex PCR for SCC*mec* typing

Multiplex PCR included eight loci (A through H) selected on the basis of *mec* element sequences described in previous reports [35]. And the primers have been described on previous reports (Table 2) [36,37]. PCR was performed on a volume of 50 mL using a Gene Amp PCR kit (Applied Biosystems, New Jersey, USA) and a kit containing the following: 1x PCR buffer II; 200 μM (each) deoxynucleoside triphosphate; 400 nM primer CIF2 F2, CIF2 R2, MECI P2, P3 MECI, RIF5 F10, RIF5 R13, R1 pUB110, and pT181 R1; 800 nM primer F2 DCS, DCS R2, P4 MECA, MECA P7 and P4 IS431; 200 nM primers KDP F1, KDP R1, RIF4 F3, and RIF4 R9; 1.25 U Ampli Taq, and approximately 5 ng of DNA template. The ASTEC program temperature control system PC-701 DNA thermo cycler was programmed as follows: 10 min at 95°C, 30 cycles of 30 seconds at 94°C, 30 seconds at 53°C, and 1 min at 72°C, and 10 minutes at 72°C. Samples were stored at 4°C until analysis. Ten-mL aliquot of the PCR products electrophoresed on 2% agarose gel (contained ethidium bromide) for 30 minutes at 100 V. Gel then photographed under ultraviolet light.

**Table 2:** **Primers used in multiplex PCR for SCC*mec *typing**

**Locus**	**Name**	**Oligonecleotide sequence (5’-3)**	**Amplicon size (bp)**	**Specificity ( *SCCmec *type)**
A	CIF2 F2	TTCGAGTTGCTGATGAAGAAGG	495	I
	CIF2 R2	ATTTACCACAAGGACTACCAGC		
B	KDP F1	AATCATCTGCCATTGGTGATGC	284	II
	KDP R1	CGAATGAAGTGAAAGAAAGTGG		
C	MECI P2	ATCAAGACTTGCATTCAGGC	209	II, III
	MECI P3	GCGGTTTCAATTCACTTGTC		
D	DCS F2	CATCCTATGATAGCTTGGTC	342	I, II, IV
	DCS R1	CTAAATCATAGCCATGACCG		
E	RIF4 F3	GTGATTGTTCGAGATATGTGG	243	III
	RIF4 R9	CGCTTTATCTGTATCTATCGC		
F	RIF5 F10	TTCTTAAGTACACGCTGAATCG	414	III
	RIF5 R13	GTCACAGTAATTCCATCAATGC		
G	IS431 P4	CAGGTCTCTTCAGATCTACG	381	pUB11C
	Pub110 R1	GAGCCATAAACACCAATAGCC		
H	IS431 P4	CAGGTCTCTTCAGATCTACG	303	III
	pT181 R1	GAAGAATGGGGAAAGCTTAC		
*mecA*	MECA P4	TCCAGATTACAACTTCACCAGG	162	Positive control
	MECA P7	CCACTTCATATGTTGTAGG		

### PCR analysis of genes related to VISA

Six genes associated with VISA strains, *vraR, vraG, vraA, vraF, fruA,*and *fruB,* were selected for PCR analysis [38]. PCR amplification was performed using primers designed from the published NCBI sequence (Table 3). PCR reaction was performed in 13 ml reaction mixture containing 10 mM Tris (pH 8.3), 50 mM KCl, 1.5 mM MgCl2, 0.2 mM each deoksinucleotide triphosphate, 0.5 mM of each primer, and 2.5 U Taq DNA polymerase (Applied Biosystems, NewJersey, USA). The ASTEC program temperature control systemPC-701 DNA thermo cycler was programmed as follows: 10 min at 95°C, 30 cycles of 1 min at 94°C, 1 min at 58°C, and 1 min at 72°C, and 10 minutes at 72°C. Samples were stored at 4°C until analysis. Ten ml aliquot of the PCR product was electrophorese don agarose gel 1.5% for 30 min at 100 V. Gels were stained with ethidium bromide and photographed under ultraviolet light. Mu50 (ATCC; 700 699) was used as a positive control.

**Table 3:** Primers used in PCR analysis of VISA-related genes

**Gene**	**Acc. NO.**	**Forward**	**Reverse**
*vraR*	ABO35448	5’-CGTCATTCAAACGGTACAAAAG-3’	5’-CTTAAAAAAGACTAAACACCAAC-3’
*vraG*	ABO35453	5’-TATTAAGGAAGGCTCACAAGTC-3’	5’-ATGTTTCAAATACCGCCCT-3’
*vraA*	ABO35450	5’-ATGAAAATGCAATAGCAGCC-3’	5’-AACATATCCTGTTGACGTCCC-3
*vraF*	ABO35453	5’-CCTCTGGATCTGGGAAAAC-3’	5’-CGTCAGCAAATATAATAGAAGGTAA-3’
*fruA*	ABO35449	5’-CTTAATGAACGGTGTTTCTAACAT-3’	5’-TACCACCAATAAATCCTGAACC-3’
*fruB*	ABO35449	5’-AGATGTTGAGTCAACTGCCTT-3’	5’-CTTCCAGCAACAATAACTATATCTTC-3’

### Ethical considerations

The project received ethical approval from the review board of the Department of National Education of the Hasanuddin University. Oral informed consent was obtained from the study participants after explanation of the procedure and the purpose of the study. Oral informed consent was applied as the collection of the specimens did not affect the intervention procedure to any extend and all clinical data was made anonymous before analysis. The collection of informed consent was witnessed by a nurse and or the medical officer in charge and was recorded on the medical file of the patient. The verbal consent procedure was approved by the ethical committee.

## Results

### MRSA and MSSA strains

Of the 11 *S. aureus* isolates from 2012 analyzed, 5 were CA-MSSA, 2 HA-MSSA, 1 CA-MRSA and 3 HA-MRSA. The 4 MRSA isolates (CA-MRSA and HA-MRSA) were derived from different clinical samples, i.e. urine, sputum, pus, and throat swab. Sensitivity testing showed that all isolates (both MSSA and MRSA) had good sensitivity to vancomycin, teicoplanin, linezolid, tigecycline, and amikacin. Clindamycin was still sensitive for CA-MSSA, HA-MSSA and CA-MRSA.

### SCC*mec* typing

**Figure 1:**
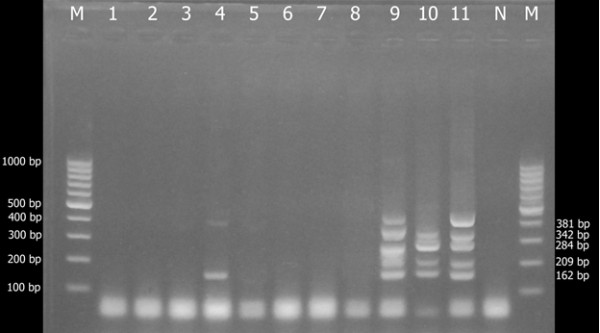
**The results of multiplex PCR SCC*mec *gene typing in MRSA and MSSA.**

SCC*mec* typing revealed that 3 of the 4 MRSA isolates contained SCC*mec* type II; the other 1 contained SCC*mec* type IV. Three of all 11 strains contained plasmid pUB110. Figure 1 shows the banding patterns of the products obtained by multiplex PCR for SCC*mec* typing. Four strains showed the 162-bp fragment of the *mecA* gene. Type II strains displayed the 284-bp fragment, 209-bpfragment, and a 342-bp fragment with or without a381-bp fragment from the plasmid pUB110; Strain type IV showed the342-bp fragment without the381-bp fragment from the plasmid pUB110.

### The presence of genes associated VISA

**Figure 2:**
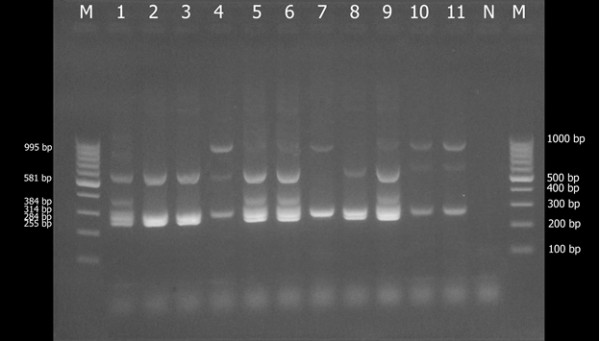
The results of multiplex PCR VISA gene in MRSA and MSSA.

The following six genes were studied: *vraR, vraG, vraA, vraF, fruA*, and *fruB*. Ten of the 11 *S. aureus* isolates contained *vraA*, 4 *vraF*, 5 *vraG,* and 4 *vraR* (Figure 2, Table 4). All 11 isolates contained *fruA* and 7 contained *fruB*. Mu50 strain was used as positive control contained all six genes. 30% of MRSA strains with pUB110 contained *vraA* genes, 60% *vraG*, 25% *vraR*, and 25% *vraF*.

**Table 4:** Characterization specimens and their related to VISA genes and pUB-110

**SPECIMENS**	**No.**	**MRSA/MSSA**	**pUB 100**	**VraA**	**vraG**	**vraF**	**vraR**	**fruA**	**fruB**
Blood	1	HA-MSSA	-	+	-	+	+	+	+
Blood	2	CA-MSSA	-	+	-	-	-	+	+
Bronchial discharge	3	HA-MSSA	-	+	-	-	-	+	+
Urine	4	**CA-MRSA**	**+**	+	+	-	-	+	-
Blood	5	CA-MSSA	-	+	-	+	+	+	+
Urine	6	CA-MSSA	-	+	-	+	+	+	+
Urine	7	CA-MSSA	-	-	+	-	-	+	-
Urine	8	CA-MSSA	-	+	-	-	-	+	+
Sputum	9	**HA-MRSA**	**+**	+	+	+	+	+	+
Pus	10	**HA-MRSA**	-	+	+	-	-	+	-
Throat swab	11	**HA-MRSA**	**+**	+	+	-	-	+	-
TOTAL	11	**11**	27%	91%	45%	36%	36%	100%	64%

## Discussion

In this study we found that three (27%) of our 11 *S. aureus* strains contained genes *mecA* and SCC*mec* type II, and 1 (9%) contained SCC*mec* type IV. Previous examined the study examined SCC*mec* types of 138 MRSA strains isolated in Japan in 1999 and found that 126 (91.3%) contained SCC*mec* type II, 6 (4.3%) contained SCC*mec* type I, and 5 (3.6%) contained SCC*mec* type IV [39]. The results of this research in Japan combined with the findings in this study suggest that type II SCC*mec* occurs frequently in Asia pacific region. Types I and III SCC*mec* were not detected in this study. However, type III SCC*mec* has been reported in European countries, Australia, New Zealand, Thailand, Vietnam, Singapore, the Philippines, and elsewhere [40,41].

In addition to the structural classification of four types of SCC*mec*, we also checked the presence of plasmid pUB110 in SCC*mec.* Only 3 strains (27%) contained plasmid pUB110. MRSA has been known to cause nosocomial infections. MRSA infections have been reported increased cases among the group of patients without any real connection with the hospital [42]. CA-MRSA strains have been reported in Australia [43,44], New Zealand [45], England [46], Canada [47], and the United States [41].

In this study we found *fruA* in all 11 strains and *fruB* in 7 of all strains (64%). Gen *vraA* considered as a long chain fatty acid CoA ligase, while *vraF* and *vraG* are ABC transporter genes. These genes are up-regulated in the VISA (Mu50) and may contribute to resistance to vancomycin [48]. Furthermore, vancomycin resistance is thought to be caused by increased cell wall synthesis [38]. The system settings are vraSR new response has been reported, and *vraR*, which is one of two components of the system, seems to play a role in vancomycin resistance. As a result of the introduction of genes into cells, *vraR* sensitive vancomycin will increase the level of resistance to vancomycin [38]. In the present study it was found that all four MRSA isolates were sensitive to vancomycin, and that one of these contained the *vraR* gene. The finding of this VISA related gene in vancomycin-sensitive among MRSA strains may indicates the possible risk of transition from MRSA to VRSA but first we need to rule out the possibility of VISA or hVISA are exist among our MRSA strain. Something lacked on this study.

Note worthily, we found that three of the four MRSA strains contained plasmid pUB110, which most likely is a strain of CA-MRSA, because it only one contained genes of *vraF* and *vraR* at relatively lower frequencies than the MRSA strains containing plasmid pUB110, but contained no genes of *vraR* and *vraF*. Therefore, we need to further investigate the relationship between SCC*mec* typing, as a means to identify the genetic background of the bacteria, as well as the presence of genes associated VISA.

## Conclusion

Most strains of MRSA: 75% (3/4) contains a Type II SCC*mec*, and only 1(25%) strain containing SCC*mec* type IV and the overall of *S.aureus* isolates containing all six genes associated VISA with different frequencies. In particular, the strain that is considered as CA-MRSA, the strain is considered to contain *vraR*, at 1 CA-MRSA strain was found not to contain *vraR* and only 33% (1/3) of *vraR* genes containing in HA-MRSA strains. Based on these results, we should be concerned about the possibility of transition from MRSA strains sensitive to vancomycin in VISA strains of MRSA strains obtained in clinical trials. But first we need to look the existence of natural VISA or hVISA among these MRSA strains.

In this study, we applied the multiplex PCR test to determine the type of SCC*mec*, and a simple PCR test to detect the presence of genes related to VISA. Both tests can be easily and rapidly performed at many hospitals and laboratories, and can therefore be considered useful tools for the investigation of clinical MRSA strains.

## Competing interests

The authors declare that they have no competing interests.

## Authors’ contributions

LB, AR and MH carried out the molecular biology studies. LB performed data and specimens collection and also epidemiology, clinical and microbiology results analysis. RD and MS participated in the molecular biology studies. All authors read and approved the final manuscript.
